# Editorial: Plant-microbes interactions and resistance against abiotic stress

**DOI:** 10.3389/fpls.2025.1599870

**Published:** 2025-04-16

**Authors:** Marzena Sujkowska-Rybkowska, Anna Rusaczonek

**Affiliations:** Department of Botany, Institute of Biology, Warsaw University of Life Sciences‐WULS, Warsaw, Poland

**Keywords:** abiotic stress, endophytes, plant-bacterial interactions, plant-fungal interactions, symbiotic microorganisms, stress-responsive genes

This Research Topic was published in late 2023. It attracted 13 papers written by 92 authors, mainly achieving our goal of providing insight into the mechanisms of plant-microbe associations and the mediation of microbes in plant tolerance to abiotic stresses. This Research Topic includes a combination of Reviews and Original Research articles covering molecular, cellular, applied, and ecological aspects of plant interactions with symbiotic microbes (rhizobia and arbuscular mycorrhizal fungi), beneficial endophytes, or plant growth-promoting (PGP) bacteria and also the role of microbes in plant resistance to various abiotic stress conditions (nutrient deficiency, osmotic stress, drought, and cold) in both the rhizosphere and phyllosphere.

## Plant-microbe interactions

Plants interact with a wide range of microbes that can modulate various aspects of plant growth and development. The review by Yang et al. provides insight into root colonization by microbes and the research gaps regarding the molecular mechanisms of plant-microbe interactions at different stages of root colonization. The Authors summarized and discussed chemotactic signals emitted by plant roots, signal reception, bacterial attachment to the root surface, bacterial immune evasion, biofilm formation and stable colonization.

Yuan et al. investigated the dynamics of the phyllosphere microbiome of pomelo (*Citrus maxima*) under changing weather parameters. The authors used Hi-Seq analysis and showed that both bacterial and fungal communities exhibited annual cycle dynamics. In another paper, Zhao et al. demonstrated how different preceding crops enhance tobacco plant growth and influence the microbial diversity and nutrient content of the rhizosphere. Preceding crops such as canola, wheat, and maize significantly increased available phosphorus, potassium, boron, and zinc in the tobacco rhizosphere. Additionally, both canola and wheat enhanced soil bacterial diversity, while wheat cultivation had the most significant impact on rhizosphere metabolite content.

The review by Verma et al. summarized the functional and mechanistic basis for the interactive role of PGP bacteria and large-scale application of nanoparticles (NPs). NPs have typically been used to enhance soil quality, while PGP bacteria have been used to stimulate plant growth and increase resistance to biotic and abiotic stresses (Kumar et al., 2017; Upadhayay et al., 2023). The Authors summarized the synergistic interactions between NPs and PGP bacteria in enhancing the soil-plant system. They presented how the negative effects of environmental factors on soil health and plant growth can be reduced by the significant potential of cooperation between PGP bacteria and NPs.

In another study, Yang et al. investigated the role of the *GmWRKY33a* gene in soybean, which is associated with brassinosteroid (BR) signaling and nodulation during symbiosis with rhizobia. BRs are phytohormones that regulate a wide range of plant developmental processes and plant interactions with different microbes (Chen et al., 2023). Notably, the Authors showed that *GmWRKY33a* is a crucial transcription factor in BR signaling and plays a negative role in nodule formation and symbiosis establishment.

## Increasing tolerance to abiotic stresses

Abiotic stress factors have unfavorable effects on plant growth and yield. Stress tolerance in plants can be enhanced by treating plants or seeds with selected microbes or synthetic compounds (Rai et al., 2021). Plant-associated microbes have the ability to alleviate stress by activating physiological, biochemical, and molecular pathways that coordinate ion uptake, nutrient metabolism, and the synthesis of compounds with osmotic or antioxidant activity (Liu et al., 2020).

Considerable interest has been shown to symbiotic fungi and PGP bacteria involved in increasing stress tolerance in plants exposed to different stressors (Kumar et al., 2017; Liu et al., 2020; Oleńska et al., 2020; Sujkowska-Rybkowska et al., 2023; Upadhayay et al., 2023). Five contributions addressed drought, which is one of the leading causes of crop losses worldwide. The mitigation of drought stress by fungi was explored in three articles. The symbiosis of desert plants (*Populus euphratica* and *Haloxylon ammodendron*) with arbuscular mycorrhizal fungi (AMF) and dark septate endophytes (DSE) was analyzed in the article by Wang et al. DSE are a group of endophytic fungi that can facilitate plant growth and stress tolerance, especially in harsh ecosystems (Li et al., 2018). The Authors showed that DSE are more dominant in extreme drought environments. Moreover, they demonstrated that AMF are more susceptible to soil factors (such as soil moisture and nutrient content), whereas DSE are more affected by pH. The article by Xu et al. focused on the enhancement of drought resistance in *Pinus tabuliformis* through ectomycorrhizal fungi and DSE application. They showed that these symbiotic fungi were able to mitigate drought stress in plants through the activation of the antioxidant system and the regulation of the osmotic balance in plant seedlings. The study by Wu et al. evaluated the impact of AMF (*Claroideoglomus etunicatum*) on tea plants under drought conditions. They showed that AMF improved tea plant adaptation to drought by enhancing the expression of N assimilation-related genes and activating related enzymes. Two studies explored the mitigation of drought stress by PGP bacteria. In another study, Kim et al. examined a novel exopolysaccharide-producing PGP bacterium, *Pseudescherichia liriopis* sp. nov. isolated from *Liriope platyphylla*, in alleviating drought and salt stress in carrot. The authors also characterized the exopolysaccharide produced by *P. liriopis*, which was shown to be an excellent antioxidant with potential practical application. The study by Park et al. investigated the drought-mitigating ability of PGP *Bacillus velezensis* GH1-13 in rice. Bacillus spp. are known for their potential and usefulness in mitigating the effects of various stresses in plants (Kumar et al., 2017). They showed that *Bacillus* enhances drought stress tolerance in rice by activating the expression of antioxidant genes and suppressing reactive oxygen species levels.

The article by Li et al. focused on cold stress and the effect of the endophytic fungus *Piriformospora indica* on the cold resistance of tobacco plants. In particular, they showed that at low temperatures, this fungus enhances the activity of the host plant’s antioxidant systems, thereby reducing oxidative stress. Furthermore, the fungus stimulates the accumulation of protective osmolytes and activates the expression of cold-responsive genes.

Finally, the study by Abd El-Daim et al. demonstrated the ability of a novel halotolerant bacterial endophyte, *Bacillus velezensis* CBE, to enhance osmotic stress tolerance in *Brachypodium distachyon* under nitrogen-deprived conditions. Moreover, they identified the molecular factors in plants that contribute to the beneficial effects of *B. velezensis* CBE in *B. distachyon* plants.

The diverse topics of the papers published in this Research Topic reflect the complexity of the interactions between plants and microorganisms in changing environments. Knowledge of the determining factors and mechanisms that regulate plant-microorganism interactions can help develop new agrobiotechnological strategies to improve plant biomass production in sustainable agriculture, as well as in soil remediation processes.
